# Changes in levels of BNP, CRP and leukocyte formula during exercise testing in patients with dilated cardiomyopathy

**DOI:** 10.1186/1749-8090-8-S1-P24

**Published:** 2013-09-11

**Authors:** V Peric, A Jovanovic, R Stolic, S Sovtic, M Sipic, P Otasevic

**Affiliations:** 1 University of Pristina, School of Medicine, Internal Clinic, Kosovska, Kosovo; 2 Dedinje Cardiovascular Institute, Belgrade, Serbia

## Background

So far, not enough clear change in biochemical parameters during exercise in patients with dilated cardiomyopathy (DCM). The aim of this paper is to study the value of BNP, CRP and leukocyte formula during exercise testing in patients with DCM.

## Methods

The study included 55 consecutive patients with DCM. All patients underwent symptoms limited exercise testing according to the Bruce's protocol. Baseline, at peak test, and 12 months after the test to all patients blood was taken for determination of these biochemical parameters.

## Results

During exercise testing have been increasing BNP (246.98 ± 571.74 vs. 257.91 ± 473.47 ng/L, p=0.002), CRP (3.83 ± 5.04 vs. 4.14 ± 5.25 mg/L, p=0.016) and WBC count (8.01 ± 1.83 vs. 10.61 ± 2.54x109/L, p<0.001). Increased the number of neutrophils (4.80 ± 1.42 vs. 5.85 ± 1.89x109/L, p<0.001), lymphocytes (2.30 ± 0.72 vs. 3.50 ± 1.15x109/L, p<0.001), eosinophil (0.20 ± 0.16 vs. 0.26 ± 0.24 x109/L, p<0.001), monocytes (0.65 ± 0.22 vs. 0.88 ± 0.29 x109/L, p<0.001) and basophils (0.01 ± 0.04 vs. 0.04 ± 0.01 x109/L, p<0.029). Twelve months after the exercise test found significantly higher BNP levels (163.93 ± 266.05 vs. 279 ± 416.58 ng/L, p=0.002) , whereas CRP (3.93 ± 5.28 vs. 3.03 ± 2.82 mg/L) and WBC (8.05 ± 1.92 vs. 7.84 ± 1.81 x109/L) did not change significantly in comparison to baseline values.

## Conclusions

During exercise testing in patients with DCM there was a significant increase in the level of BNP, CRP, and all parameters of leukocyte formula.

